# Telemedicine in post–CABG patients: promises and pitfalls

**DOI:** 10.1007/s12471-021-01537-y

**Published:** 2021-01-21

**Authors:** L. Hofstra, G. A. Somsen

**Affiliations:** Cardiology Centres of the Netherlands, Amsterdam, The Netherlands

Escalating healthcare costs and the aging population, combined with a surge in eHealth technology, have opened up a novel space in healthcare delivery [1, 2]. The current COVID-19 crisis has highlighted the need for telemedicine and has accelerated efforts to implement eHealth solutions [3]. Telemedicine may be especially useful in high-demand, low-resource settings [2].

Roughly, telemedicine in cardiology can be divided into three different domains [4]. First, a combination of sensors and smartphone apps allows for the detection of arrhythmia in patients under a variety of circumstances and for a prolonged period of time. Preliminary data from our *HartWacht* programme demonstrate high patient satisfaction and a reduction in visits to the emergency departments. Second, the chronic management of diseases such as diabetes mellitus, heart failure and hypertension may benefit from the use of wearable devices that communicate via smart technology with platforms, which in turn deliver advice based on algorithms and/or specialised nurses via video consulting [5]. Third, telemedicine could have a significant impact on improving lifestyle and guiding rehabilitation programmes by using a combination of wearable technology (f.e. step-counting device) and automated advice on diet, exercise et cetera [6].

Telemedicine requires efficient handling of the data by determining individual thresholds and specific algorithms. Central (automated) interpretation of these data is increasingly recognised as a requirement for cost-effective and scalable telemedicine programmes. In addition, as with any innovation in healthcare, implementation and reimbursement are still major hurdles in the widespread adoption of telemedicine in cardiology.

With this framework in mind, the IMPROV-ED trial offers a chance to prove the incremental value of telemedicine in the first weeks following coronary artery bypass grafting (CABG) [7]. The readmission rate and unplanned consultations of patients in the first weeks after CABG is relatively high. This trial offers an opportunity to study the addition of teleconsulting and educational videos on the reduction of readmissions and unplanned consultations. The strength of the study design is the randomised approach, which will provide the most reliable scientific answer as to whether telemedicine in patients undergoing CABG has added value with respect to healthcare utilisation and quality of life [8]. Given a positive outcome, the fact that the study is randomised will help convincing policymakers and health insurance companies to provide reimbursement for telemedicine for the post–CABG patient.

A potential pitfall of the IMPROV-ED trial is the power calculation, which is based on reports of readmission and unplanned use of care in post–CABG patients in the United States. Hannan et al., as cited by the authors, showed that readmission predominantly occurs in the most vulnerable patients [9]. The question is whether these patients are able to adopt the novel approach. In addition, given the scope of possibilities available for telemedicine in cardiology, the approach that was chosen by the IMPROV-ED investigators to use educational videos and video consulting, seems narrow [4]. The addition of technology, for instance, to monitor arrhythmias, blood pressure and body temperature could have strengthened the study. In addition, telemedicine is only scalable if irrelevant data are (automatically) filtered out for physicians. This should be addressed because this is a prerequisite for a cost-effective eHealth programme.

A low-cost telemedicine intervention following CABG could be of special significance in these settings. We performed a meta-analysis of the cost-effectiveness of telemedicine programmes and identified the following critical factors for successful implementation: (a) focus on high intensity interventions, (b) involve a large number of participants, (c) and use telemedicine as a (partial) replacement for usual care [10]. The IMPROV-ED study is a first step towards validation of telemedicine in post–CABG patients and may serve as a landmark study, contributing to the economic and medical acceptance of telemedicine.
